# Integrating the promotion of physical activity within a smoking cessation programme: Findings from collaborative action research in UK Stop Smoking Services

**DOI:** 10.1186/1472-6963-10-317

**Published:** 2010-11-25

**Authors:** Adrian H Taylor, Emma S Everson-Hock, Michael Ussher

**Affiliations:** 1 School of Sport and Health Sciences, University of Exeter, Heavitree Road, Exeter, EX1 2LU, UK; 2 Division of Community Health Sciences, St. George's University of London, Cranmer Terrace, London, SW17 0RE, UK; 3 Current Address: Section of Public Health, School of Health and Related Research, University of Sheffield, Regent Court, 30 Regent Street, Sheffield, S1 4DA, UK

## Abstract

**Background:**

Within the framework of collaborative action research, the aim was to explore the feasibility of developing and embedding physical activity promotion as a smoking cessation aid within UK 6/7-week National Health Service (NHS) Stop Smoking Services.

**Methods:**

In Phase 1 three initial cycles of collaborative action research (observation, reflection, planning, implementation and re-evaluation), in an urban Stop Smoking Service, led to the development of an integrated intervention in which physical activity was promoted as a cessation aid, with the support of a theoretically based self-help guide, and self monitoring using pedometers. In Phase 2 advisors underwent training and offered the intervention, and changes in physical activity promoting behaviour and beliefs were monitored. Also, changes in clients' stage of readiness to use physical activity as a cessation aid, physical activity beliefs and behaviour and physical activity levels were assessed, among those who attended the clinic at 4-week post-quit. Qualitative data were collected, in the form of clinic observation, informal interviews with advisors and field notes.

**Results:**

The integrated intervention emerged through cycles of collaboration as something quite different to previous practice. Based on field notes, there were many positive elements associated with the integrated intervention in Phase 2. Self-reported advisors' physical activity promoting behaviour increased as a result of training and adapting to the intervention. There was a significant advancement in clients' stage of readiness to use physical activity as a smoking cessation aid.

**Conclusions:**

Collaboration with advisors was key in ensuring that a feasible intervention was developed as an aid to smoking cessation. There is scope to further develop tailored support to increasing physical activity and smoking cessation, mediated through changes in perceptions about the benefits of, and confidence to do physical activity.

## Background

Evidence-based smoking cessation services provided by the UK National Health Service (NHS) utilise a combination of behavioural support and pharmacotherapy [1] and increase 12-month quit rates from 2-5% unaided to 15-20% [2,3]. Cessation advisors come into contact with approximately 500,000 UK smokers attempting to quit each year (using Stop Smoking Services), probably resulting in about 13,000 smokers who permanently quit [4]. Given the throughput of clients, even small additional increases in cessation rates would provide worthwhile public health gain.

Intervention studies to examine the effects of physical activity on smoking cessation have shown mixed results, due to methodological limitations and possibly because they have largely involved structured exercise programmes involving 30-60 mins of moderate - vigorous intensity physical activity on 2-3 occasions per week, with a focus on increasing fitness and weight management [5]. Only one study (in the UK) involved brief (i.e., approximately 5 mins per session) individual physical activity counselling in addition to standard behavioural and pharmacological treatment, over 7 sessions, but failed to show a significant positive effect on smoking cessation [6,7]. Also, a physical activity intervention, involving the use of a pedometer at 12 and 20 weeks post-quitting increased cessation rates [8].

Given the evidence that a single session of short bouts of low-moderate intensity physical activity reduces cravings and withdrawal symptoms, and cue-reactivity [9], during temporary abstinence, it would appear that this could be translated into new integrated smoking cessation interventions. Indeed this has been done with isometric exercise, translating the findings from a study on the acute effects in a laboratory study [10] to delivery in an intervention [11]). Interviews with advisors suggest that simultaneous health behaviour change (ie, healthy eating and physical activity) in conjunction with smoking cessation is possible for some quitters [12] but smoking cessation advisors in the UK are generally trained to avoid the promotion of multiple behaviour change at the time of quitting [1]. No research to date has examined how promoting physical activity, specifically as a cessation aid integrated into standard behavioural and pharmacological treatment, may support cessation, especially in a group-based cessation programme.

There is clearly some interest in the use of physical activity to support smoking cessation. A national survey [13] found that 56% of 170 smoking cessation advisors promoted physical activity in their NHS clinics. Among the whole sample, they reported spending, on average, 29 minutes promoting physical activity over a 6/7-week clinic, with 55% promoting it for weight and craving management. In a national survey of 181 clients [14] attending a Stop Smoking Service, 22% reported currently using physical activity to control their smoking, and 35% had used it during a previous quit attempt. Further, clients had relatively positive beliefs that physical activity was a useful aid for smoking cessation, with a mean rating of 4.4, on a - 3 to + 3 scale.

### How should physical activity best be promoted?

The challenge then is to develop an integrated intervention that (a) advisors can deliver without it depreciating a focus on quitting using standard behavioural and pharmacological support, (b) clients find complementary, rather than burdensome and irrelevant, at the time of quitting and beyond, (c) promotes broader health gain among people with often a cluster of poor lifestyle behaviours, and (d) helps prevent weight gain which often contributes to relapse.

In a national survey of advisors [13], those who promoted physical activity held more positive beliefs regarding pros and cons, self-efficacy, outcome efficacy and importance of physical activity as a smoking cessation aid. Of those who did not promote physical activity, over 40% felt that they had insufficient time or a lack of expertise, while only 18% of advisors thought it would be unpopular among clients. In a national survey of smokers [14], those using physical activity as a cessation aid held more positive beliefs regarding self-efficacy to do physical activity and outcome efficacy (i.e., greater utility). The identification of these theoretical constructs provides a basis from which to develop an intervention to support advisors and in turn smokers in their use of physical activity as a cessation aid. However, we were also acutely aware that advisors hold considerable experience in working with clients, with respect to multiple health behaviour change [12], and their input into the design of an intervention was critical.

Collaborative action research is a useful method for refining and evaluating interventions. This approach typically involves "a process though which practitioners (insiders) are encouraged to review and alter aspects of practice by an 'outsider'" [15]. Action research involves "a period of inquiry that describes, interprets and explains social situations while executing a modified intervention aimed at improvement and involvement." It is "problem-focused, context-specific and future-oriented" [16]. In the present study, the authors (the 'outsiders') encouraged the smoking cessation practitioners, who were keen to enhance the number of clients who successfully quit smoking, to integrate physical activity promotion as a cessation aid into their normal practice.

In order to integrate and evaluate the promotion of physical activity in an existing Stop Smoking Service a two-step feasibility study was conducted. In Phase 1, the aim was to develop an integrated intervention, embedded within current standard pharmaceutical and behaviour smoking cessation support, to promote physical activity as a cessation aid through a process of collaborative action research. In Phase 2 the aims were as follows: (1) to pilot a training programme for smoking cessation advisors; (2) monitor the integrated programme delivered to smokers attending a clinic within NHS Stop Smoking Services. Within Phase 2 we used field notes, interviews and collected survey data on (1) smoking cessation advisor beliefs and behaviour with respect to promoting physical activity, and (2) client beliefs and behaviour with respect to using physical activity as a cessation aid, as part of a feasibility study to determine issues associated with implementation of the intervention. It was not a trial to determine effects on smoking cessation, nor was it necessarily powered to detect changes in the survey measures. Thus this work fitted within Phase 1 (development) and 2 (pilot/feasibility) of the Medical Research Council (MRC) Guidelines for developing complex interventions [17], in that we sought to identify key multiple components in developing the intervention, and used mixed methods to assess any relevant changes in advisor and client behaviour related to those components. Such work leads to a more refined intervention, to be delivered within a pilot and potentially a subsequent fully powered trial to determine intervention effectiveness.

## Methods

### Procedures

The study was approved by the local NHS Research Ethics Committee. All participants gave written informed consent.

A summary of the methods for Phase 1 and 2 is shown in Table 1. More detailed information on the participants, and data collection and procedures is reported below.

**Table 1:** Summary of methods in developmental and evaluation phases

	Participants	Data collection	Data analysis
Phase 1	3 advisors in Plymouth PCT (South West England).	Field notes and reflective diary. Observation of clinics and discussions with advisors, throughout 3 cycles of collaborative action research	Identified pros and cons of each adaptation of the intervention, with advisors.

Phase 2 (Aim 1)	7 advisors in South Birmingham PCT (Midlands) working with groups in clinics. 1 advisor in Plymouth working with individuals.	Surveys of advisors' physical activity promotion actions, beliefs, and personal characteristics, before training and after implementation. Field notes, reflective diary, interviews and discussions with advisors regarding their beliefs and progress.	Paired t tests to compare pre and post training and intervention delivery.

Phase 2 (Aim 2)	111 clients took a self-help guide. 72 clients (32% male) (15 of whom were seen individually in Plymouth) completed T1 surveys, but only 27 at T2.	Surveys of quitters' use of physical activity, beliefs, and personal characteristics. Field notes, reflective diary, interviews and discussions with advisors regarding clients' beliefs and progress.	Paired t tests to compare pre and post the 6/7 week Stop Smoking Clinic intervention, using intent to treat (i.e. all those completing survey at T1).

An NHS Stop Smoking Service was initially selected involving staff who were interested in working with the researchers and also engaged with smokers from a mixed socio-economic background. In Phase 1, the intervention was refined through three cycles of collaboration involving three sequential group clinics. Initially, in the first cycle, the authors were introduced to the clients by the advisors as collaborative researchers seeking to enhance the effectiveness of the clinic through the promotion of physical activity. The researchers initially led a 15 min session on physical activity for smoking cessation at the end of a standard group-based clinic, with the aid of some key points on a one page handout. There was some repetition of content (e.g., discussion of coping with withdrawal symptoms) and in subsequent cycles the intervention became more integrated and entirely delivered by the advisor. Within each cycle, reflection (discussion on content and materials), planning (revising objectives, materials and content) and implementation (delivery of adapted intervention) took place and involved both the researchers and advisors. At the end of these cycles, a self-help guide for clients was produced to take forward into Phase 2.

We had intended to extend Phase 1 into Phase 2 in the same NHS Stop Smoking Service but group clinics were suspended, forcing us to seek a new partner. We advertised through GLOBALink, a Smoking Cessation practitioner research network for collaborators in the UK, which led us to work with the South Birmingham NHS Stop Smoking Service. Of several Services who offered support, this one ensured involvement with a mixed socio-demographic population.

As shown in Table 1, Phase 2 involved an evaluation of the effects of a training programme and intervention delivery on advisor behaviour and cognitions about promoting physical activity in a clinic (Aim 1), and the effects of the 'Walk-2-Quit' intervention on client physical activity behaviour and related cognitions (Aim 2).

### Intervention

The content of the two hour advisor training session is shown in Table 2. This was deliberately kept relatively brief for pragmatic reasons and we wished to determine if such training could make a difference to the delivery of a standard clinic. The training programme also prepared advisors to use pedometers (Yamax, Digi-Walker) in conjunction with the self-help guide, which had additional pages for self-monitoring of daily steps. Table 3 shows a mapping of the respective intervention components, aims, contents, and process and outcomes expected to be changed. We continued to make minor changes to the intervention over four sequential waves of clinics, in response to feedback from the advisors who led the clinics, as shown in Table 4.

**Table 2:** Structure and content of Walk-2-Quit training

Duration (total 2 hours)	Aims	Content
15 mins	Review evidence on physical activity intervention content and effectiveness.	1. Summary of findings from 12 chronic studies. 2. Summary of interventions used (structured exercise limited counselling) 2. Discuss practical implications.

15 mins	Review evidence on acute effects of physical activity for managing cravings.	1. Summary of findings from 20 acute studies. 2. Discuss practical implications of using physical activity for mood and craving management.

15 mins	Review evidence on weight management strategies in smoking cessation	1. Summary of findings from studies on weight gain during smoking cessation. 2. Discuss advisor strategies used to prevent weight gain.

15 mins	Introduce aims of Walk-2-Quit within the context of a standard clinic (and NHS training)	1. Outline aims and content of Walk-2-Quit, relative to traditional pharmacological and behavioural support. 2. Highlight cognitive and behavioural processes associated with changing clients' use of physical activity as an aid.

30 mins	Train advisors to integrate physical activity promotion into a cessation clinic over 6/7 weeks.	Highlight use of self-help guide (from week 1-6/7) to identify key weekly strategies to increasing use of physical activity for managing cravings, emotional eating and weight.

15 mins	Train advisors to use pedometers for self-monitoring physical activity for mood and craving regulation.	Highlight use of pedometers and other strategies for behavioural and emotional regulation. Link to self-help guide and spaces for weekly self-monitoring.

15 mins	Summarise and review implementation of Walk-2-Quit	1. Identify advisor concerns and level of confidence to change current practice to a more integrative approach to multiple behaviour change. 2. Review Walk-2-Quit content to further enhance advisor beliefs about the benefits and personal role in targeting client beliefs.

**Table 3:** 'Walk-2-Quit' intervention components, aims and content

Intervention component	Aim	Content	Process and outcome evaluation
Use client-centred approach in clinic.	Develop rapport with client, building trust, and shared respect.	Effective communication skills. Exhibit empathy, listen, reflect, summarise.	Clients individually able to talk about their physical activity and smoking experiences.

Elicit beliefs about quitting and physical activity as a behavioural strategy.	Increase self-awareness and build confidence to quit, using behavioural and pharmacological support.	Clients identify pros & cons of quitting and aids for quitting. Focus discussion on physical activity through direct and vicarious experiences of clients.	Clients identify physical activity as a promising behaviour to aid smoking cessation.

Cognitive processes to promote physical activity as a smoking cessation aid, alongside other aids.	Increase pros and reduce cons for physical activity and increase self-efficacy and outcome efficacy.	Facilitate client discussions to introduce physical activity as a behaviour that is not just structured exercise, and helps to manage cravings, withdrawal symptoms & weight.	Clients increase beliefs in physical activity as a coping strategy and aid to quitting.

Behavioural processes to increase physical activity as a smoking cessation aid, alongside other aids.	Develop behavioural strategies to increase physical activity.	Set SMART goals with clients to increase physical activity. Think about timing of physical activity, mood and avoiding lapses. Signpost to physical activity/exercise opportunities & remove barriers to do physical activity. Elicit social support for physical activity.	Self-monitoring used (e.g. pedometer worn and physical activity diary kept). Rewards and reinforcement contingencies established.

Review and reflect on increased physical activity.	Build confidence and perceptions of control, & ability to self-regulate mood and cravings with physical activity.	Client reflects on physical activity successes and sets new targets. Identify identity shifts (e.g. smoker to exerciser). Highlight physical activity related mood and well-being.	Client increases confidence to do more physical activity and stay quit.

**Table 4:** Minor changes made to the intervention during Phase 2

Change made	Reason for change	Effect of change
Self-help guides for using physical activity as an aid also included information on pharmaco-therapies.	Advisors and clients requested information on available pharmaco-therapies previously given out on separate handouts to be added to self-help guide.	The distribution of fewer leaflets to clients was welcomed.

The pedometer goal of 10,000 steps was amended to a 10% increase in steps per week following the first wave of clinics.	Some less active clients became despondent about being able to achieve 10,000 steps per day.	Less active clients more motivated to make progressive changes, giving advisors an opportunity to give tailored guidance to individuals.

An advisor guide and index was produced that was cross-referenced to the weekly content of the self-help guide.	Advisors wanted an easier weekly guide to follow.	Some advisors found this very useful, others did not find it necessary.

An additional 7-day daily step count diary was developed and distributed with the SHGs.	Advisors reported that their clients wanted to be able to record their steps every day and see the pattern over a week.	Some clients used this form, whereas others did not.

Isometric exercises were added to the SHG.	Advisors reported some client interest in this, especially for those who could not go for a walk while at work.	A few clients found this helpful, however an audio file would have been more helpful.

### Data collection

Self-reported age, gender, smoking history, height, weight and 7-day physical activity recall [18] were assessed for all advisors and clients.

Phase 1: Field notes and reflective diaries were maintained from April 2006 to September 2006.

Phase 2: The seven advisors who provided much of the NHS Stop Smoking Service in South Birmingham completed surveys prior to receiving training to deliver 'Walk-2-Quit' and after delivering the next 7 week intervention for the first time. Following training, advisors invited all group-based clients to complete a survey at the first session of their respective 7-week clinics. Those who were still attending at the end of the clinic (i.e. 4 weeks post-quit) were also invited to complete a second survey. No attempt was made to assess clients who were not at the final session, due to a lack of resources and a common assumption that clients without confirmed CO abstinence at 4 weeks post-quit had relapsed [19]. Surveys at baseline and follow-up sought to collect data on changes in behaviour and beliefs and also pilot the use of data collection materials.

To assess advisor beliefs about physical activity as a smoking cessation aid, multi-item measures, adopted from a previous survey [13] were used. Items focused on stage of readiness to promote physical activity as a cessation aid, beliefs about the pros and cons, self-efficacy, outcome efficacy and importance of promoting physical activity.

To assess client beliefs about physical activity as a smoking cessation aid, multi-item measures adopted from a previous survey [14] were used. Items focused on stage of readiness to use physical activity as a cessation aid, self-efficacy to do 30 mins of moderate intensity physical activity on most days of the week, and beliefs about the expected benefits of physical activity as a cessation aid.

### Data analysis

In Phase 1, an iterative process evolved in which the pros and cons of each aspect of the intervention were explored with advisors. These were recorded and appropriate adaptation of the intervention took place in the next cycle of collaborative action research [20].

In Phase 2, data from the surveys were analysed using SPSS Version 14, for both advisors and clients. Scores on sub-scales, as reported previously [13,14] were created. Pre-post (T1-T2) comparisons were made on all variables using paired samples *t*-tests, for both seven advisors and the clients. Comparisons for clients are reported for those completing data at baseline and end of clinic, and also using an intent to treat analysis, as a more conservative approach to examining change. As such, for clients who did not complete a follow-up survey (i.e., 54 of 72, or 75%) we imputed their baseline scores. It was not possible to determine whether failure to provide data at T2 was due to failure to return to the clinic due to relapse to smoking or unwillingness to complete the survey as a successful quitter after four weeks.

## Results

Across the two phases of the study the 11 advisors had a mean (SD) age of 44.7 (11.4) years, 8 were female, and they reported spending an average (SD) of 41.4 (28.5) minutes over the course of a typical 6/7-week clinic promoting physical activity, prior to the study. The advisors all had many years of experience in smoking cessation work.

### Phase 1

Across the three cycles of collaborative action research several key findings emerged: (1) 'Walk-2-Quit' became quite distinct from previous practice; (2) The self-help guide emerged from a single information sheet (in cycle 1) to become a key feature by the end of cycle 3, involving a guide with 30 pages of practical ideas (but theory-driven) on how to become more physically active with a specific aim to help manage weight, mood and cravings; (3) 'Walk-2-Quit' had shifted the focus from smoking cessation to an holistic approach to a healthier lifestyle, and helped focus group discussions on physical activity as a cessation aid (but not an additional behaviour per se); (4) all the usual information and support provided by advisors (e.g., pharmacological and social) remained but physical activity became a topic that emerged throughout the 6-week programme, from prior to quitting to the final session, four weeks after quitting; (5) the final cycle of observation, reflection, planning and implementation took place while a 6-week clinic took place in a particularly socially deprived area of the city. Several lessons were learnt from this. First, clients, especially with physical disabilities, found use of the self-help guide more difficult. Advisors took additional care to keep the physical activity options simple, and drew on others in the group to highlight how to individually overcome barriers to increase physical activity and seek enjoyable and sustainable low cost options. It was also very evident that collecting any information (e.g. surveys) on physical activity behaviour and beliefs was especially challenging.

### Phase 2

In Phase 2, the 7 advisors in South Birmingham had a mean (SD) age of 48 (7.4) years (range 39 - 58 years), 6 were female, three didn't achieve public health guidelines of 150 mins per week of moderate or vigorous intensity physical activity (overall mean mins (SD) was 208 (114), and mean (SD) BMI was 25.1 (2.8)(range 22 - 30). They reported spending about 6 mins over the course of a typical 7-week clinic promoting physical activity, at the start of the study.

In Phase 2, the 72 clients who completed assessments at the start of the intervention, had a mean (SD) age of 47.0 (11.3) years, a BMI of 26.3 (5.5), self-rated health (1 - 5 scale, where 5 = excellent) of 2.7 (0.8) and smoked 19.3 (11.3) cigarettes per day prior to the quit attempt.

### Intervention feasibility

Several observations relating to the feasibility of the intervention were made in the Phase 2 clinics. We report these under the headings of behavioural, psychological, intervention process and evaluation process issues. Comments are linked to specific advisors in South Birmingham (SB) and Plymouth (P).

### Behavioural issues

The pedometer appeared to provide clients with a behavioural focus. The advisors generally reported that clients seemed more focused on their walking and pedometer steps than on the self-help guide. Advisor SB1 reported that one client in an early clinic had made an effort to double their steps, however the pedometer served as a behavioural focus in another way; advisor P3 reported that many clients found it useful to fiddle with it when they went out for a walk, where they would previously have fiddled with their cigarettes and lighters. Advisor P3 reported that one client who was fearful of weight gain found it useful to count steps as a method of increasing activity. Another advisor found on chance encounters that some clients would still be wearing the pedometer three months after the clinic (SB2).

The advisors also reported that clients would discuss their use of physical activity in general as a behavioural strategy during their quit attempt. Advisor SB3 reported people getting off the bus a stop earlier, using the stairs instead of the lift, engaging in daily walking and buying and using a trampoline, whereas advisor SB1 reported that one client had tried the isometric exercises (added to the self-help guide before the second round of clinics) but found it difficult without an audio file. It was also reported that some clients found the intervention useful, whereas others found it more difficult due to poor mobility, and that those clients who had begun to engage in physical activity continued to do so throughout the duration of the clinic (SB3).

Interestingly, delivering this intervention also had an impact on advisor behaviour, both in terms of their own physical activity levels and habits and also in terms of an interest in health promotion training. Some advisors had tried out the pedometers and reported being more active as a result (SB1, SB2, SB5). Advisor SB1 reported that many advisors took to wearing a pedometer on most days and one advisor reported losing a significant amount of weight during this time. Advisor SB5 had been inspired by the intervention to do more health promotion work and training.

### Psychological issues

Throughout the intervention process, the advisors reported few negative client perceptions about the pedometers. Advisor P3 reported that a small number of clients didn't want to use them or didn't find them helpful, however most clients "love the pedometers" and one client was "buzzing with it". Advisor P3 also suggested that clients liked it as it focused them on what they did each day, with some clients being surprised at how much they did each day at work: They saw it as "fantastic" and "a tool - a very positive tool", "giving them something they want and like". Advisor P3 reported that one client went from doing 3000-4000 steps a day to doing 6000-7000 steps a day over three weeks, because the client was "really liking it and using it". Advisor SB1 reported that some of her clients felt disheartened by the '10,000 steps a day' message and addressed this despondency by promoting a general increase in step count instead. Advisor SB3 reported that "clients mentioned steps and how the counter was a motivator" and advisor P3 also found her clients motivated by knowing their number of steps, which in turn became self-competitive.

While the pedometers provided an opportunity for self-monitoring of physical activity, in particular walking and lifestyle activity, one advisor (SB1) felt that increases in physical activity involved more of a subconscious process than goal oriented behaviour. Advisor SB1 suggested that although it was obvious in group discussions that clients had been using physical activity to help them, and were increasing their physical activity, clients often appeared to be unaware of these increases until group discussions in the clinic.

Similarly, advisors SB1 and SB3 noted that clients often expressed surprise that they had been using physical activity in the way suggested and were also sometimes surprised that physical activity had actually been helping them deal with cravings and withdrawal symptoms.

In general, advisors found that clients did not talk about how physical activity made them feel (SB1, P3), but talked about the step count and were generally positive about doing it and about the link between walking/exercise and quitting smoking (P3, SB3). Clients did not seem to discuss the barriers to increasing physical activity (e.g. cost, time) that are normally raised in conjunction with promoting structured exercise programmes, which was an important objective of the intervention.

### Intervention process issues

In most clinics, the group discussion format was primarily used as a forum for physical activity promotion and active clients were proactively sought to lead. For example, advisor SB3 reported that the dialogue of the clients who found physical activity to be useful seemed to influence others in the group.

Introducing physical activity from the start of the clinic (rather than several weeks after quitting, as was previously the norm) was useful as clients then expected to discuss physical activity as part of the quitting process, including in workplace groups. Advisor SB2 noted, "clients have no problem talking about physical activity right from the very first week in a group discussion, perhaps because it's a workplace group and they already know each other pretty well."

The self-help guide seemed useful for both clients and advisors but the extent of client engagement varied. One advisor reported having some good discussions using the guide in the first round of clinics, as many people brought it to the group, however in the second round of clinics this wasn't the case: "clients don't always bring the self-help guides into the session, but it's obvious that they've read them, as they often say things like, 'in the book it says this...'" (SB1). Another advisor suggested that clients found discussions around some elements of the guide to be useful (SB3). Advisor P3 found that where clients brought the guide in they had been using the charts to record their quit success and lapses, although on the whole they didn't seem to read the self-help guide and rarely completed the diary asking about their feelings. Often, advisors would report a mixed reaction, whereby some clients found the self-help guide useful while others preferred to focus more on the pedometer. Indeed one barrier to using the guide was time, especially for completing the diaries (SB1, SB2, SB3, P3). Advisors found the self-help guides useful in delivery for planning sessions and focusing the discussion (SB1, SB2, SB4, SB5). Two advisors recalled spending time specifically discussing the various sections in the self-help guide during the sessions (SB1, SB3) and one advisor reported distributing the self-help guide to clients for general information instead of the usual information pack, as it was sufficiently comprehensive (P3).

In terms of delivery, the advisors generally reported that the intervention was easily integrated into their usual practice and was not an excessive workload. One advisor reported being initially worried, as it was something new, but found it really easy to integrate (P3) and another commented, "without the (survey) forms it would be almost effortless to deliver" (SB1). Indeed, the data collection forms appeared to form the bulk of the workload (see next section), and two advisors expressed an interest in using the intervention (without the data collection forms) as part of their usual delivery (SB1, P3). Advisor SB1 expressed an interest in integrating this intervention into different smoking cessation settings such as working with mental health patients, drop-in sessions and clinics for pregnant and obese smokers, and was interested in training the whole service to deliver the intervention. Similarly, advisor P3 had already trained a colleague who had recently started work to deliver the intervention and was keen to obtain pedometers and continue to run a similar scheme, suggesting that it could be a useful tool to address the needs of clients and advisors in the context of relapse rates (P3).

### Evaluation process issues

The primary issue with the evaluation was that the data collection added considerably to advisors' workload and many commented that it would have been easier without it (SB1, SB2, P3). Chasing up data collection forms that had been taken home was time consuming (P3), whereas offering clients the opportunity to complete the forms in the sessions was easier and clients were happy to do this (SB1, SB2).

### Preliminary quantitative findings

The main purpose of the study was to develop the intervention and assess its feasibility, and as such the study was not powered to detect a pre-post difference in behaviour or beliefs about the use and usefulness of physical activity. Nevertheless, some preliminary quantitative findings are summarised in Tables 5 and 6. These findings tentatively suggest that the intervention has some potential to change advisor and client behaviour and beliefs, although further research with adequate power is needed to quantitatively assess the impact of the intervention on both advisors and clients. Both time spent promoting physical activity by advisors and their self-efficacy to promote physical activity in clinics significantly increased. Of the 22 clients who provided data on minutes of moderate and vigorous physical activity at baseline and end of the seven week clinic, 4 decreased, 11 increased and 7 stayed the same. A Wilcoxon Signed Ranks Test revealed no significant change (p = 0.31), based on negative ranks, but the median minutes of moderate and vigorous physical activity more than doubled.

**Table 5:** Preliminary quantitative data for effect of intervention on advisors

Outcome	n	T1 mean (SD) score	T2 mean (SD) score	*t *value	*p *value	95% confidence intervals
Total time allocated to promoting PA (mins)*	7	41.9 (28.5) (median = 40)	64.3 (33.7) (median = 70)	2.2	0.06	-2.5 to 48.2

Self-efficacy for promoting PA	7	7.1 (1.8)	8.4 (0.9)	2.6	0.04	0.1 to 2.7

Outcome expectancy	7	7.8 (2.2)	8.3 (1.2)	0.8	0.44	-1.0 to 2.1

Pro beliefs	7	5.7 (0.9)	5.8 (0.8)	1.7	0.13	-0.0 to 0.3

Con beliefs	7	2.5 (0.7)	1.9 (0.7)	-2.1	0.08	-1.1 to 0.1

Importance of promoting PA	7	6.4 (1.1)	7.0 (0.0)	1.3	0.23	-0.5 to 1.6

PA = Physical Activity

* Over a 7 week clinic

**Table 6:** Preliminary quantitative data for effect of intervention on clients

Outcome	n	T1 mean (SD) score	T2 mean (SD) score	*t *value	*p *value	95% confidence intervals
Stage of readiness to use PA as a cessation aid (1-5)	23	2.7 (1.1)	3.4 (1.6)	3.76	0.001	0.4 to 1.2

Stage of readiness to use PA as a cessation aid (intent to treat) (1-5)	72	2.3 (1.1)	2.6 (1.3)	3.34	0.001	0.1 to 0.4

Outcome expectancy (1-7)	22	5.8 (0.8)	5.9 (1.3)	0.23	0.819	-0.5 to 0.6

PA levels (mins/wk moderate & vigorous PA)	22	143.4 (274.5) (median = 42.5)	162.5 (202.1) (median = 102.5)	0.22	0.826	-446 to 553

Self-efficacy for smoking cessation (1-7)	24	4.7 (1.5)	5.9 (1.2)	3.03	0.006	0.4 to 2.0

Self-efficacy for dealing with stress (1-7)	24	4.3 (1.7)	5.1 (1.6)	2.81	0.010	0.2 to 1.4

Self-efficacy for doing 30 min PA on most days (1-7)	24	4.8 (2.1)	5.2 (2.1)	1.48	0.153	-1.7 to 1.0

Self-efficacy for walking briskly for 15 min (1-7)	24	4.9 (2.0)	5.3 (2.1)	1.40	0.175	-1.8 to 0.9

PA = Physical Activity

## Discussion

Through consecutive cycles of collaboration and reflection, the intervention progressed from a separate physical activity component delivered by researchers to a comprehensive and integrated strategy to promote physical activity as a cessation aid. It incorporated a comprehensive self-help guide and pedometers, which involved promoting physical activity in the 2 weeks prior to quitting and 4 weeks after quitting. The smoking cessation advisors were enthusiastic about implementing the refined version of the intervention, quickly increased their confidence to promote physical activity (based on survey responses and field notes), and reported that clients had started using physical activity as a way of dealing with situational cues, cravings and withdrawal symptoms. The pedometers were particularly popular, both as a motivational tool and as a form of distraction.

Our finding that the reaction to the pedometers was generally positive is consistent with previous research in a variety of populations. For example, sedentary individuals with type 2 diabetes engaged in a pedometer-based intervention liked the pedometer as a self-monitoring tool [21], ethnic minority populations reported enthusiasm for pedometer use [22], and midlife women found the pedometer functioned as a motivational tool [23]. Similarly, our finding that some clients were disheartened by the 10,000 steps goal was consistent with previous research that has found that the goal may be unrealistically high for sedentary people or those with chronic diseases and can lead to attrition from research and reduced adherence to pedometer-based programmes [24]. This goal was also found to be unpopular among middle-aged men in Australia [25], although a meta-analysis suggested that a step goal such as 10,000 steps was an important predictor in increasing physical activity levels [26]. While the pedometers in the present study were popular, it was a challenge to get clients to self-monitor their daily steps using the recording sheets in the self-help guide. Prochaska and colleagues [8] also reported that less than 15% of their sample completed 6 weeks of data sheets, recording pedometer steps, to facilitate smoking cessation. Finally, our finding that the advisors found it possible to promote physical activity right from the start of the clinic is consistent with previous research that found the simultaneous promotion of multiple health behaviours changes to be more effective than the sequential promotion of multiple changes [27] despite a common contrary belief [1].

The training and delivery of the intervention did not significantly impact on advisors' stage of readiness for promoting physical activity as a smoking cessation aid, outcome efficacy beliefs or pro and con beliefs. This is perhaps not surprising, given the small sample involved. Another explanation may be that advisors already had favourable scores on these measures towards physical activity, and comparison with data from a national survey suggests that this was the case [13]. Such advisor selection bias was unavoidable as we wished to collaborate with a Stop Smoking Service that was interested in promoting physical activity at this stage in our development, implementation and evaluation of 'Walk to Quit'. However, time spent promoting physical activity over a typical 6/7-week clinic and self-efficacy for promoting physical activity in smoking cessation clinics increased from before the training to the end of the delivery of the first clinic. Thus, the process of conducting the intervention (and undergoing associated training) appears to have raised the profile of physical activity promotion in smoking cessation clinics, indicating feasibility of this strategy, albeit without a parallel control group. Further research, with adequate power, is needed to explore if training can impact on the professional practice of advisors who have less favourable beliefs about physical activity as a smoking cessation aid.

Ussher and colleagues [6] reported that about 5 mins of counselling, in addition to usual care, increased quitters' physical activity. In our study, advisors reported an increase from about 6 to almost 10 mins per week in physical activity counselling, which could lead to even greater increases in quitters' physical activity, with hypothetically greater effects on smoking cessation. Ussher had a separate block of time to promote physical activity to individual quitters who had been recruited for a study on the effects of exercise on smoking cessation. In contrast 'Walk-2-Quit' is optimally fully integrated into a whole clinic. This may create problems in recalling when and how much physical activity counselling took place, and more objective observational research is needed to code advisor - client interactions to identify physical activity promoting behaviours and client discussion on physical activity to overcome this limitation.

The intervention impacted upon some client behaviour and beliefs in the anticipated direction, namely stage of readiness to use physical activity as a cessation aid, self-efficacy for smoking cessation and self-efficacy for dealing with stress during the quit attempt. However, it is possible that the latter two outcome effects are a function of the smoking cessation programme rather than 'Walk-2-Quit' per se, and these findings should be interpreted with caution in the absence of a control condition. Future parallel controlled studies with adequate power would ascertain the extent to which such an effect could be attributed to physical activity promotion.

The intervention did not appear to statistically change our measure of clients' mean self-reported levels of physical activity (though clearly there were increases in the median score). This may have been due to the small sample size. It could also be due to the nature of the physical activity being promoted. Sporadic lifestyle physical activity, perhaps useful for temporary management of controlling smoking urges and withdrawal symptoms is less easy to recall and report on a survey than sessions of more intense longer-duration structured physical activity designed for fitness gain. Thus it may be that either the quitters did not consider such sporadic bouts as being 'physical activity' (i.e. a measurement error), or the total amount of activity taken for controlling urges to smoke was not sufficient to raise the total amount of physical activity recorded using the 7-day physical activity recall survey. Further research, controlled and adequately powered, is needed to assess the impact on physical activity using accelerometers (not pedometers) to detect more subtle changes in physical activity. The intervention also did not significantly impact upon clients' self-efficacy for physical activity or outcome expectancy beliefs regarding physical activity as a cessation aid. A longer-duration or more intense/more belief-focused physical activity promotion strategy may be required to impact on these variables, if indeed they are important mediating variables. When the baseline data was compared with a larger national sample from a cross-sectional survey [14], the clients in the current study had stronger self-efficacy beliefs for being regularly active, weaker self-efficacy beliefs for quitting smoking, did less moderate physical activity in the previous week, and were slightly older. The extent to which the characteristics of the present sample impacted on the findings is unclear, but again, a ceiling effect could explain the lack of change in self-efficacy for physical activity.

Whereas a structured exercise programme may require greater planning and inconvenience, the present study and previous work [12] suggests that short bouts of physical activity could be promoted at the same time as a quit attempt, with less propensity for 'cognitive overload' for quitters. Indeed, brief bouts of physical activity were seen as a positive coping behaviour that could naturally diminish the urge to smoke and snack [28-30], and could be promoted by advisors for craving self-regulation, particularly during the first four weeks after quitting. This fits well with a recently developed multiple affective behaviour change approach (e.g. [31,32]), in which the promotion of regular brief bouts of physical activity may regulate mood, which in turn reduces desire to smoke and sugar snack, and engage in other mood regulating behaviours.

During the time of the intervention implementation and evaluation (January to August 2008), 2464 clients accessed the South Birmingham Stop Smoking Service, 46% of whom were verified, with standard CO monitoring, as being quit at 4 weeks. We had very mixed success in recruiting clients through advisors, rather than a designated researcher as is common in research trials, despite frequent contact with the advisors. Comments from the advisors suggested that they were aware of the additional burden of clients completing surveys at the beginning and end of each clinic, and that this may have limited participation. In addition, some advisors reported that the length of the surveys could be off-putting to some clients. However, examining the feasibility of delivering and evaluating the intervention among smokers being treated (rather than recruits into a study) was one of the strengths of the present study.

Clients in the study joined respective Stop Smoking Service clinics unaware of the 'Walk-2-Quit' intervention and evaluation, until asked to give consent and complete baseline evaluation forms. While self-selection bias may have taken place at this point we are confident that the intervention was delivered in a contextually generalisable setting, at least within NHS Stop Smoking Services. In comparison with our published national survey of clients attending a Stop Smoking Service [11] and advisors [10], clients in the present study were comparable on age, sex, BMI, cigarettes per day before current quit attempt and times quit smoking in the last year, but did more vigorous physical activity, less moderate physical activity and were less ethnically diverse. Advisors were comparable on BMI, sex and smoking history, but did less vigorous and more moderate physical activity, were slightly older, spent more time promoting physical activity and were less ethnically diverse.

## Conclusions

While there are recognised challenges in facilitating multiple health behaviour change within smoking cessation in general and promoting physical activity in smoking cessation clinics specifically (e.g. how to time multiple changes, work with mixed abilities and/or clients with special needs in Stop Smoking Service groups), our data suggest that this is certainly possible and could potentially be undertaken on a wider scale. In the light of emerging evidence to support a role for multiple health behaviour change in smoking cessation [8], there is a need for the training of smoking cessation advisors to attempt a more integrated approach towards promoting a positive behaviour rather than largely focusing on avoidance of a negative behaviour. Given that the considerable weight gain that follows smoking cessation impacts on relapse, it appears to be an unreasonable option to not promote physical activity (and healthy eating) at the time of quitting for many clients [33].

It is evident that the standard 6- or 7-week smoking cessation clinic is likely to remain focused on evidence-based pharmacological and behavioural therapies until further scientific support is provided for the effectiveness of physical activity as an aid. However, it is also clear from the present study that it is feasible to promote physical activity as an aid to smoking cessation. Collaboration with advisors was key in ensuring that a feasible intervention was developed. There is scope to further develop support that is more tailored to individual needs and beliefs with respect to increasing physical activity and smoking cessation. A pilot randomised trial appears justified to test the effectiveness on smoking outcomes and process measures of weight gain and physical activity, in the context of a rigorous, adequately powered trial with data collection prior to, during and after the 'Walk-2-Quit' intervention compared with usual care. The intervention could be evaluated in both group and individually delivered formats. This form of physical activity intervention is distinctly different from others that have predominantly focused on promoting vigorous bouts of longer periods of exercise (e.g. up to 60 mins) for 2-3 times per week in conjunction with an abrupt attempt to quit smoking (e.g. [34]). Our intervention may be more attractive to quitters who are typically less physically active and tend to avoid vigorous exercise, and who may find the initiation of a structured exercise programme, at the same time as quitting, to be too mentally challenging. Our intervention also has some common elements with emerging interventions designed for one-to-one support [35].

## Competing interests

The authors declare that they have no competing interests.

## Authors' contributions

AHT designed the overall study. AHT and ESE-H designed the intervention and drafted the manuscript, engaged in collaboration with the Stop Smoking Service, collected the data and conducted the analyses. MU helped to draft the manuscript and contributed to the study design and data analysis. All authors read and approved the final version of the manuscript.

## Pre-publication history

The pre-publication history for this paper can be accessed here:

http://www.biomedcentral.com/1472-6963/10/317/prepub
